# Ginsenoside Rg5 alleviates hypoxia-induced myocardial apoptosis by targeting STAT3 to promote Tyr705 phosphorylation

**DOI:** 10.1186/s13020-025-01128-8

**Published:** 2025-06-13

**Authors:** Fang-yang Li, Yi-hao Wang, Cheng Zhang, Wan-yun Dang, Ze-kun Wu, Zhen-hui Wu, Jia-lu Cui, Xiang-jun Wu, Chun-qi Yang, Xue-cong Tian, Cheng-rong Xiao, Yu-guang Wang, Yue Gao

**Affiliations:** 1 School of Pharmacy, Guangdong Pharmaceutical University, Guang Zhou, 510006 China; 2 Beijing Institute of Radiation Medicine, Beijing, 100850 China; 3 School of Pharmacy, Jiangxi University of Chinese Medicine, Nanchang, 330000 China; 4 Department of Nephrology, First Medical Center of Chinese, PLA General Hospital, Nephrology Institute of the Chinese People’s Liberation Army, National Key Laboratory of Kidney Diseases, National Clinical Research Center for Kidney Diseases, Beijing Key Laboratory of Kidney Disease Research, Beijing, 100853 China; 5 School of Computer Science and Technology, Xinjiang University, Urumqi, Xinjiang, 830046 China

**Keywords:** Ginsenoside Rg5, Stat3, Tyr705 phosphorylation, Hypoxia, Myocardial apoptosis

## Abstract

**Background:**

The heart, as the body's blood-pumping organ, is extremely sensitive to changes in oxygen levels. Myocardial injury caused by hypoxia is a challenging issue, and there are currently no definitive specific drugs available for its treatment. Ginsenoside Rg5, one of the main rare saponins in ginseng, has shown significant efficacy in treating myocardial injury. This study aims to investigate the role and mechanisms of Rg5 in the treatment of hypoxic myocardial injury.

**Methods:**

The cardioprotective effect against acute hypoxia of Rg5 was studied by assessing heart function, myocardial injury markers, inflammation, and oxidative stress in C57 mice, as well as apoptosis and reactive oxygen species (ROS) levels in H9c2 cardiomyocytes. Thermal proteome and target validation techniques were used to confirm the target protein of Rg5. The further protective mechanisms against hypoxia-induced damage were explored using immunocoprecipitation, immunofluorescence and rescue experiments in vivo and in vitro.

**Results:**

The experimental results demonstrated that Rg5 effectively improved cardiac function in mice, reduced inflammation, oxidative stress, and the release of myocardial injury markers, decreased cardiomyocyte apoptosis, and lowered ROS levels. Further, using target protein screening and validation techniques, Signal transducer and activator of transcription 3 (STAT3) was verified as a direct target for Rg5's cardioprotective effect. It was observed that Rg5 specifically promoted the phosphorylation of Tyr705 in STAT3 via the JAK2/STAT3 pathway, leading to the translocation of phosphorylated STAT3 into the nucleus where they induce the expression of anti-apoptotic protein and protect cells from hypoxic damage.

**Conclusion:**

Rg5 could be a potential therapeutic agent for preventing and treating myocardial hypoxic injury, providing scientific evidence for its application in anti-hypoxic therapy.

## Introduction

Due to convenience in transportation and economic development, people can travel quickly from low to high altitudes. However, drastic changes in the environment can bring great challenges to the human body. Acute mountain sickness, such as headaches, palpitations and shortness of breath, often occurs when the individuals rapidly ascend from low-altitude to high-altitude areas [1]. In the case of long-term hypoxia, it can cause diseases such as cardiac remodeling and myocardial hypertrophy, seriously endangering human health [2, 3]. The heart increases its pumping efficiency by increasing beat rate and strengthening myocardial contractility, thereby ensuring that various tissues receive as much oxygen and nutrients as possible in high-altitude environments [4]. Hypoxia can cause irreversible damage to cardiomyocytes and even lead to myocardial death [5]. Due to the non-reproducibility of cardiomyocytes [6], even if oxygen supply is subsequently restored, it will not be able to recover the already damaged cardiomyocytes. Existing studies have shown that apoptosis is one of the main damages to cardiomyocytes under hypoxic conditions [7], in which excessive oxidative stress and inflammation are important pathways of hypoxia-induced myocardial apoptosis [7–11].

Ginseng (*Panax ginseng* C.A. Meyer), a time-honored medicinal plant, boasts a rich history of use, especially in treating myocardial diseases where it has proven excellent efficacy. Its medicinal effects stem largely from its diverse chemical composition, including saponins/ginsenosides, polysaccharides, phenolic compounds, volatile oils, alkaloids, and proteins. Currently, pharmacological research on ginseng mainly focuses on ginsenosides. Ginsenoside Rg5, a unique saponin derivative from ginseng, has garnered considerable attention due to its outstanding biological activity. Rg5 demonstrates a wide range of therapeutic effects, including anticancer, anti-inflammatory, antioxidant, anti-hypoxic, and cardioprotective properties [12]. Specifically, Rg5 can effectively reduce hypoxia-induced cardiomyocyte damage by inhibiting cell apoptosis, thereby enhancing the resistance of cardiomyocytes to ischemic injury [13]. Additionally, it improves mitochondrial function, further increases the survival of cardiomyocytes under ischemic conditions [14].

Signal transducer and activator of transcription 3 plays a crucial role in various physiological and pathological processes. STAT3 not only participates in fundamental biological processes such as cell proliferation, differentiation, and survival but also plays an important role in immune regulation, inflammatory responses, and tissue repair. In the heart, the high expression and active state of STAT3 make it as a crucial molecule for maintaining cardiac function and shielding the myocardium from damage [15, 16]. The activation of STAT3 primarily depends on two critical phosphorylation sites: tyrosine 705 (Y705) and serine 727 (S727). When cytokines (such as IL-6) or growth factors (such as EGF) bind to their receptors, they activate receptor-associated Janus kinases (JAKs), which then phosphorylate the Y705 of STAT3. Phosphorylated STAT3 binds to specific DNA sequences to regulate the expression of target genes. The phosphorylation of S727 can be mediated by various kinases (such as MAPKs, PKC, etc.), and it works together with the phosphorylation of Y705 to regulate the activity of STAT3 [17, 18]. This signaling pathway is vital in various physiological and pathological processes, especially in the heart, where the activation of the JAK2/STAT3 signaling pathway can reduce cardiomyocyte apoptosis and enhance cardiac function [19, 20].

In this study, we confirmed the pharmacological effect of ginsenoside Rg5 on alleviating acute hypoxic myocardial apoptosis through in vivo and in vitro experiments. At the same time, we used a variety of molecular technical approaches to reveal that Rg5 can directly target STAT3 and enhance the interaction between STAT3 and JAK2, inducing the phosphorylation at tyrosine 705 of STAT3. This research indicated that Rg5 could be a cardioprotective drug against acute hypoxia.

## Materials and methods

### Animals and experiment protocol

Twenty-four male C57BL/6 mice (20—22 g), 6 weeks old, were supplied by Charles River Laboratories (Beijing, China). All animal care and experimental procedures in this study were approved by the Animal Ethics Committee of the Academy of Military Medical Sciences (No: IACUC-DWZX-2023-P685). All procedures strictly followed the National Institutes of Health Guidelines for the Care and Use of Laboratory Animals. Before starting the in vivo experiments, the mice were given 7 days to acclimate to their surroundings. The mice were housed under a 12:12 light–dark cycle and provided with typical rodent food.

The mice were randomly divided into 4 groups: Control, Hypoxia, Hypoxia + Ginsenoside Rg5, and Hypoxia + Ginsenoside Rg5 + Stattic (n = 6). Ginsenoside Rg5 was dissolved in a 2.5% sodium carboxymethyl cellulose solution and administered orally at a dose of 30 mg/kg for 7 days. Stattic was administered via intraperitoneal injection for 7 days at a dose of 5 mg/kg. After a 7-day period of drug administration, the three hypoxic groups of mice were placed in a low-pressure hypoxia simulation chamber (DYC-9070, Fenglei Aerospace & Ordnance Co., Ltd., Anshun, China) for 3 days, which was set to mimic an altitude of 6500 m (≈28.04 kPa). And The altitude rise and fall speed inside the cabin is 5 m/s. During this period, the mice had normal access to water and food. After the simulation ended, the simulated altitude was lowered to 3500 m, and the researchers entered the simulation chamber to collect samples from the mice.

### Echocardiography

Using the 6 LAB animal ultrasound (Beijing Yeeran Technology Co., Ltd.), equipped with a French VERMON high-frequency probe, to record mouse echocardiography. M-mode and 2-D parasternal long-axis scans at the level of the papillary muscles are conducted in order to assess changes in Ejection Fraction EF (%) and Fractional Shortening FS (%).

### Enzyme-linked immunosorbnent assay (ELISA)

The levels of serum Interleukin-1 beta (IL-1β), Interleukin-6 (IL-6), Tumor Necrosis Factor-alpha (TNF-α), Superoxide Dismutase (SOD), Malondialdehyde (MDA), Glutathione (GSH), Cardiac Troponin I (cTnI), Myoglobin (MYO), Creatine Kinase (CK), and Creatine Kinase-MB (CK-MB) were measured using ELISA kits provided by Jiangsu Meimian Co., Ltd. (cat:MM-0040M2、MM-0163M2、MM-0132M2、MM-0389M2、MM-0897M2、MM-0661M2、MM-0791M2、MM-0518M2、MM-0734M2、MM-43703M2) (Jiangsu, China).

### Heart histology

After anesthetizing the animals, the heart tissue was removed from the thoracic cavity and fixed in 4% paraformaldehyde. Heart tissues were sectioned (5 mm thick), embedded in paraffin blocks, and stained with hematoxylin–eosin (HE) for morphological examination.

### Cell cultures and treatment

H9c2 cells (rat cardiomyocytes) were obtained from Procell (CL-0089, Wuhan, China). The cells were grown in DMEM supplemented with 10% fetal bovine serum (FBS), 100 μg/mL streptomycin, and 100 μg/mL penicillin and maintained at 37 °C in a humidified atmosphere of 5% CO_2_.

Before the experiment, the drugs were diluted in DMEM containing 2% fetal bovine serum (FBS) as the diluent. The cultured H9C2 cells were then placed in Bugbox M instrument (Forward, Suzhou, China) for 24 h to undergo hypoxic treatment (5% CO_2_, 1% O_2_). For the drug treatment groups, ginsenoside Rg5 was administered at concentrations of 0.25, 0.5, and 1 μM for 24 h before the hypoxic exposure. Additionally, stattic (5 μM) and colibelin (1 μM) were administered 4 h before the hypoxic exposure.

### Cell counting kit-8 assay

H9c2 cells were seeded into 96-well plates at a density of 3 × 10^4^ cells per well and maintained at 37°C with 5% CO_2_ for 24 h. Cell proliferation was assessed using the Cell Counting Kit-8 (CCK-8, TargetMol, Shanghai, China) according to the manufacturer's instructions. Absorbance was measured at 450 nm using a microplate reader.

### Apoptosis detection

To differentiate apoptotic cells from viable or necrotic cells, a combined staining of annexin V-FITC and PI was used (Elabscience Biotechnology Co.,Ltd,Wuhan China). To study the damage caused by hypoxia on H9_C_2 cells, the treated cells were trypsinized, washed with PBS, and resuspended in annexin-binding buffer. The cell suspension (1 × 10^5 cells/mL) was then incubated with FITC-labeled annexin V and PI in the dark for 20 min. Cell counting was performed using BD FACSAria™ III (USA), and the data were analyzed with FlowJo version 10.8 software. A total of 10,000 cells per sample were recorded. Fluorescence spectra of annexin V and PI were detected using 527/32-nm and 586/42-nm filters, respectively.

### JC-1 mitochondrial transmembrane potential assay

The mitochondrial membrane potential was assessed using the fluorescent probe JC-1 dye (Elabscience Biotechnology Co.,Ltd,Wuhan China). Cells processed during the experiment were harvested by trypsinization, washed with PBS, and resuspended. The cell suspension (1 × 10^5 cells/mL) was then prepared. Quantification of the number of mitochondria with green (JC-1 monomers) and red (JC-1 aggregates) fluorescence was performed using emission filters at 535 nm and 595 nm, respectively. The immunolabeled cells were analyzed using a BD FACSAria™ III flow cytometer.

### Determination of reactive oxygen species

H9_C_2 cells were trypsinized and then treated with 500 nM DCFH-DA (Beyotime Shanghai China) probe at 37°C for 20 min, followed by two washes with PBS. The fluorescence intensity of DCFH-DA was measured using a BD FACSAria™ III flow cytometer with an excitation wavelength of 488 nm. Data analysis was performed using FlowJo version 10.8 software.

### Separation of nuclear/cytoplasm protein fractions

According to the manufacturer's instructions, the cytoplasmic and nuclear fractions of H9_C_2 cells after treatment were isolated using a commercial Nuclear and Cytoplasmic Extraction Kit (P0027, Beyotime, Shanghai, China). Protein concentration was determined using a BCA Protein Assay Kit.

### Seahorse analysis

H9_C_2 cells were analyzed using the Seahorse XFe96 Analyzer (Agilent Technologies) for real-time measurement of oxygen consumption rate (OCR). Briefly, cells (3 × 10^4 cells/well) were seeded onto a Seahorse 96-well cell culture microplate (Agilent Technologies). Cells were then treated with different concentrations of Ginsenoside Rg5 for 24 h. Following treatment, cells were subjected to hypoxic conditions for 24 h in Bugbox M instrument (5% CO_2_, 1% O_2_). Subsequently, the medium was changed to non-buffered, glucose-free DMEM supplemented with 2 mM glutamine. The Seahorse XF Cell Mitochondrial Stress Test Kit (103,708–100, Agilent Technologies) was used according to the manufacturer's instructions.

### Mitochondrial transition pore assay

According to the manufacturer's instructions, Mitochondrial Permeability Transition Pore Assay Kit of MPTP Assay Kit (Beyotime, Shanghai, China) was used to assess the generation of mitochondrial superoxide in live H9c2 cells following treatment with the drug and hypoxia.

### Measurement of mitochondrial superoxide generation

According to the manufacturer's instructions, MitoSOX™ Green mitochondrial superoxide indicator (Thermo Fisher Scientific, Massachusetts, USA) was used to assess the generation of mitochondrial superoxide in live H9c2 cells following treatment with the drug and hypoxia.

### Immunofluorescence staining

H9c2 cells were grown on coverslips for 12 h and then exposed to the treatment. After treatment, the cells were washed three times with PBS and then fixed with 4% paraformaldehyde at room temperature for 15 min. Following fixation, the cells were rinsed with immunofluorescence wash buffer and then blocked with immunofluorescence blocking buffer at 4°C for 2 h. The coverslips were then incubated overnight at 4°C with the primary antibody against p-STAT3 (Tyr705) (#9145, CST). Subsequently, the coverslips were incubated with a fluorescent secondary antibody for 1 h. Finally, the slides were mounted using an anti-fade mounting medium containing DAPI. The slides were then examined using a confocal microscope (Nikon, Tokyo, Japan).

### Western blotting

H9_C_2 cells were lysed on ice for 30 min in RIPA lysis buffer containing 1 mM protease inhibitors and 1 mM phosphatase inhibitors(GRF101、GRF102, Yamei, Shanghai, China). The lysate was then centrifuged at 3500 rpm for 10 min at 4°C, and the supernatant was collected. Protein concentration was determined using a BCA protein assay kit. Equal amounts of protein were loaded onto Bis–Tris gels for SDS-PAGE separation. Proteins were transferred onto PVDF membranes, which were incubated overnight at 4°C with primary antibodies, including p-STAT3 (Tyr705) (#381,552, Zenbio), p-STAT3 (Ser727) (#9134, CST), STAT3 (#10,253–2-ap, Proteintech), JAK2 (YT2426, Immunoway), cleaved caspase-3 (#9661, CST), cleaved caspase-9 (#9509, CST), Cytochrome c (#11,940, CST), Bcl-2 (YT0470, Immunoway), and Bax (#YT0449, Immunoway).

### Cellular thermal shift assay (CETSA)

H9c2 cells were collected in PBS buffer and the whole cell proteins were extracted by repeated freeze–thaw cycles. The cell lysates were then incubated with DMSO (1%) or Rg5 (0.5 μM) on ice for 1 h. The mixture samples were divided into eight equal portions and incubated in parallel at different temperatures ranging from 42°C to 66°C for 3 min. After incubation, the samples were centrifuged, and the supernatants were used for Western blot analysis to detect the presence of STAT3.

### Drug affinity responsive target stability (DARTS) assay

H9c2 cells were collected and resuspended in PBS. Proteins were extracted by repeated freeze–thaw cycles, followed by centrifugation at 3500 rpm for 15 min at 4°C. The lysate was divided into five equal aliquots and treated with DMSO and different concentrations of Ginsenoside Rg5 (0.25, 0.5, and 1 μM) on ice for 1 h. After incubation, proteolysis was carried out on ice for 15 min using a protease (HY-B2228, MCE, China) (enzyme: cellular protein = 1:500). Finally, 5 × loading buffer was added to denature the proteins, and the expression of STAT3 was detected by Western blotting.

### Immunoprecipitation (IP)

The H9c2 cells were transfected with a Flag-tagged STAT3 plasmid purchased from MiaoLing Plasmid Platform(Hubei, China) using a transfection reagent. After the completion of the drug experiment, the STAT3 and its interacting proteins were extracted from the transfected H9c2 cells using the Flag-Tag Protein Immunoprecipitation Assay Kit with Agarose Gel (Beyotime, Shanghai, China). The interaction was then confirmed through Western Blot analysis.

### Real-time polymerase chain reaction

Total RNA was extracted from H9c2 cells using TRIzol reagent (15,596,026, Thermo Fisher Scientific). The RNA was then reverse-transcribed into cDNA using the TransScript kit (AT311-02, TransGen Biotech, Beijing, China). qPCR reactions were performed using PerfectStart (AQ601-02-V2, TransGen Biotech) with β-actin as the internal reference to quantify the expression levels of the target genes. The forward and reverse primers for rat Bcl-2, Mcl-1 and β-actin were 5′-GAACTGGGGGAGGATTGTGG-3′, 5′-GGGGTGACATCTCCCTGTTG-3′; 5′-AACTGGGGCAGGATTGTGAC-3′, 5′-CCCGCTTCGTCCTTACAAGA-3′; 5′-CCCGCGAGTACAACCTTCTT-3′, 5′-CGCAGCGATATCGTCATCCA-3′. The primers used in this study were synthesized by Sangon Biotech (Shanghai, China).

### Thermal proteome sample preparation

H9c2 cells were lysed and incubated with 0.5 μM Rg5 for 30min, and then incubated at 37°C and 60°C for 3 min, followed by 3 min at room temperature. Then 2 × SDS loading buffer was mixed with whole cell lysates incubated at different temperatures. After centrifugation, soluble protein concentration was determined by BCA method, and then an equal amount of protein samples was taken for in-gel digestion as described previously [21]. In order to reduce the influence of SDS-PAGE gel particles on the capillary column, the extracted peptides were further purified by a homemade C18 Stage-Tip. Finally, the cleaned peptide mixtures are vacuum-dried for mass spectrometry analysis.

### LC–MS/MS analysis

Thermal proteome sample were analyzed on an Orbitrap Exploris™ 480 Mass Spectrometer (Thermo Scientific) after LC separation (Vanquish Neo, Thermo Scientific). The peptides mixtures were firstly loaded onto a Trap capillary column (75 μm × 2 cm), then nonlinear separated by a long analytical capillary column (75 μm × 25 cm). The gradient of LC is: 1–6% B for 4 min, 6–31% B for 138 min, 31–95% B for 2 min, 95% B for 6 min (Buffer A, 0.1% FA in ddH_2_O; Buffer B, 20% ddH_2_O and 0.1% FA in ACN). The MS survey scans were collected at 60,000 resolution in the 350–1200 m/z range, Cycle time 2s and Normalized AGC target 300%; for MS/MS analysis: fragment ions were recorded with a resolution of 15,000, Cycle time was set to control the number of MS2 count per cycle. The raw files were searched by Proteome Discoverer software (v3.0) against Swiss-Prot reviewed rat library (2024–06). Semi-cleavage by Trypsin was set and 1% FDR was controlled at PSM, peptide and protein levels. A total of six raw was analyzed to quantify protein content in two groups. For algorithms incompatible with missing data, the KNN method was employed to impute missing values [22]. The differentially expressed proteins (DEPs) were screened using Bioconductor package limma [23] with criteria of | log_2_ (fold change) |> 0.58 and P value < 0.05. Stat3 peptide spectrum matching information was obtained from Proteome Discoverer software and then visualized by pFind software [24].

### Molecular docking

Stat3 structure information was retrieved from the RCSB protein database. PyMol software (version 1.8) and AutoDockTools software (version 1.5.6) were used to make modifications such as isolating original ligands, removing water molecules, adding hydrogens, and repairing amino acids. Molecular docking was then performed using Autodock-vina (version 1.1.2). The docking results were visualized through PyMol software and docking interaction patterns were established.

### Statistical analyses

The significance of the data was handled in GraphPad Prism 8 (GraphPad Software, La Jolla, CA). We employed the Student’s *t*-test when comparing two groups and one-way ANOVA followed by Tukey’s multiple comparison test for more than two groups. Statistical significance was determined at P < 0.05.

## Results

### Ginsenoside Rg5 relieves myocardial injury caused by hypoxia exposure

**Fig. 1:**
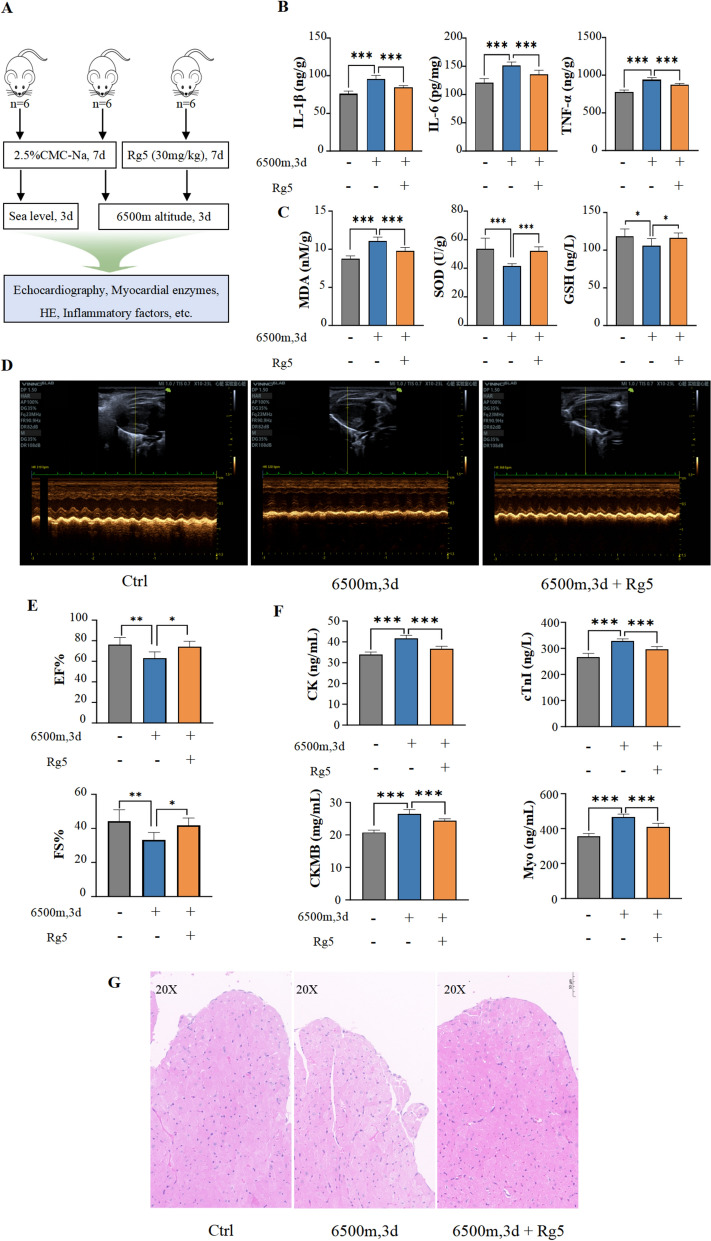
Rg5 alleviated myocardial damage caused by acute hypoxia in vivo **A.** Schematic diagram of animal experiment procedure. A total of 18 mice were divided into three groups: Ctrl group, Mod group and Rg5 group. The Mod and Rg5 groups were exposed at 6500 m altitude for three days. **B.** Content changes of IL-1β, IL-6, and TNF-α in peripheral blood (n = 6). **C.** Changes of MDA, GSH and SOD activity in peripheral blood (n = 6) **D.** Representative echocardiographic image of mice in three groups. **E.** Rg5 improved EF, ejection fraction and FS, left ventricular fractional shortening of mice exposed to hypoxia (n = 6). **F.** Content changes of myocardial injury markers in the peripheral blood. CK, Creatine kinase; cTnI, Cardiac troponin I; CK-MB, Creatine kinase-MB; Myo, Myoglobin (n = 6). **G.** Representative HE staining image of heart tissues in three group of mice (n = 3).. Scale bar = 50 μm. Data are shown as mean ± SD. *P < 0.05, **P < 0.01, ***P < 0.001 as indicated

**Fig. 2:**
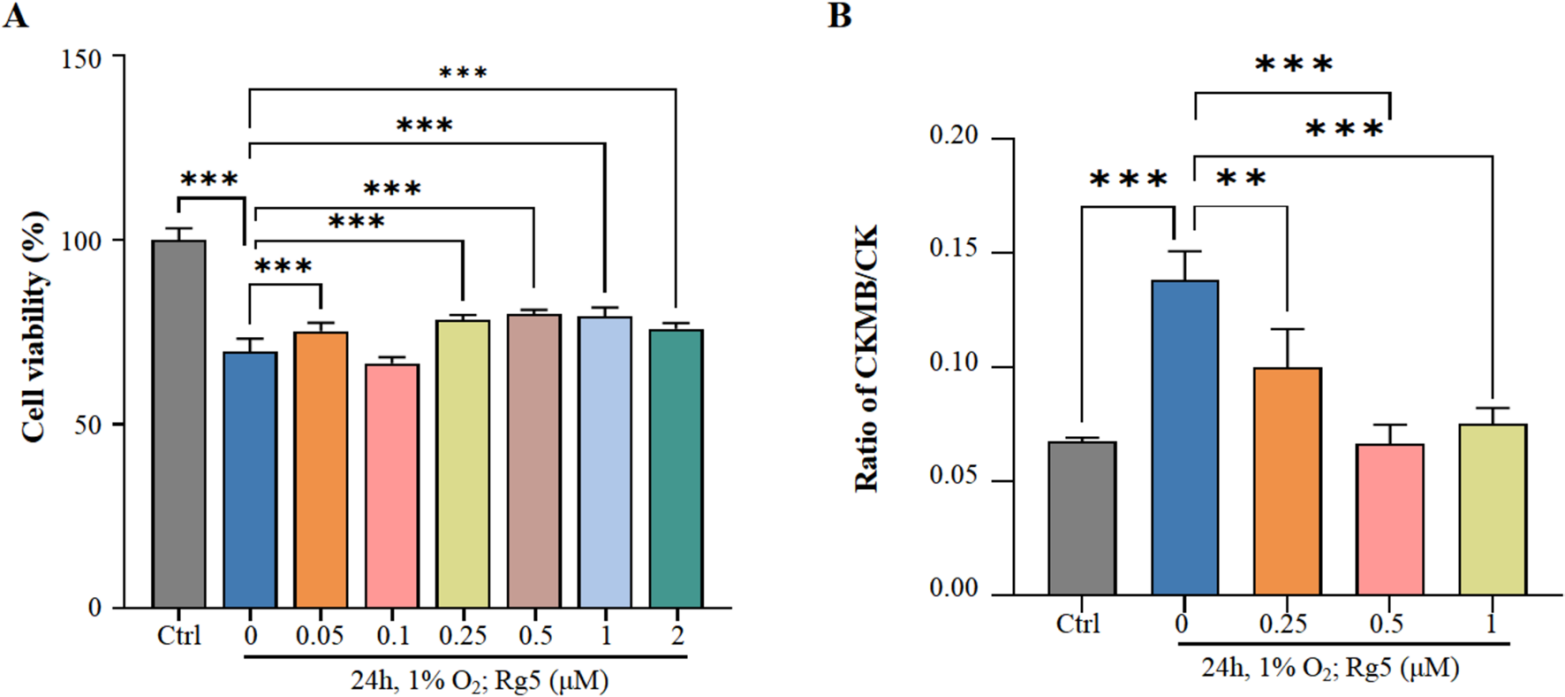
Rg5 improved H9c2 cardiomyocyte injury induced by acute hypoxic exposure **A.** Rg5 increased the cell viability of H9c2 cardiomyocytes after hypoxic exposure (n = 6). **B.** The content ratio of CKMB/CK in H9c2 culture medium supernatant was significantly reduced after Rg5 treatment (n = 6). Data are shown as mean ± SD. *P < 0.05, **P < 0.01, ***P < 0.001 as indicated

To study the effects of Rg5 on acute hypoxic myocardial injury, we utilized a low-pressure hypoxic environment simulation chamber (6500 m illustrated in Fig. 1A) and a cellular anaerobic chamber (1% O_2_) to simulate acute hypoxia in mice and H9c2 cells, respectively. From the observed indicators, hypoxia led to an increase in the body's inflammatory response and oxidative stress, whereas pre-intervention with Rg5 could mitigate inflammation and oxidative stress injuries in mice (Fig. 1B, C). Compared with the Ctrl group without hypoxia exposure, the ejection fraction and fractional shortening of mouse heart in Mod group were significantly reduced. However, administration of Rg5 could restore myocardial function during hypoxia (Fig. 1D, E). The detection of myocardial enzymes and myocardial injury markers showed that pre-administration of Rg5 could effectively ameliorate related indicators (Fig. 1F), indicating that Rg5 could reduce the myocardial injury caused by hypoxia exposure. Additionally, using H&E staining, the pathological changes in myocardial tissue were detected under a light microscope. No significant abnormalities were observed in the Ctrl group, whereas in the group with hypoxia treatment, hypertrophy of myocardial endocardial cells, widened intercellular spaces, and vacuoles in some cells were noted. Treatment with Rg5 significantly improved these pathological damages (Fig. 1G). In vivo, the cell viability test results showed that Rg5 (0.25 µM, 0.5 µM, 1 µM) could enhance cell survival under hypoxic conditions (Fig. 2A). The detection of myocardial enzymes from the culture medium supernatant of H9c2 cells also indicated that cellular damage was less in the Rg5 group compared to the group with hypoxia treatment (Fig. 2B). This result was consistent with the aforementioned findings, suggesting that Rg5 had a protective effect on the myocardium during hypoxia exposure.

### Rg5 alleviates oxidative stress and mitochondrial damage in myocardial cells caused by acute hypoxia

**Fig. 3:**
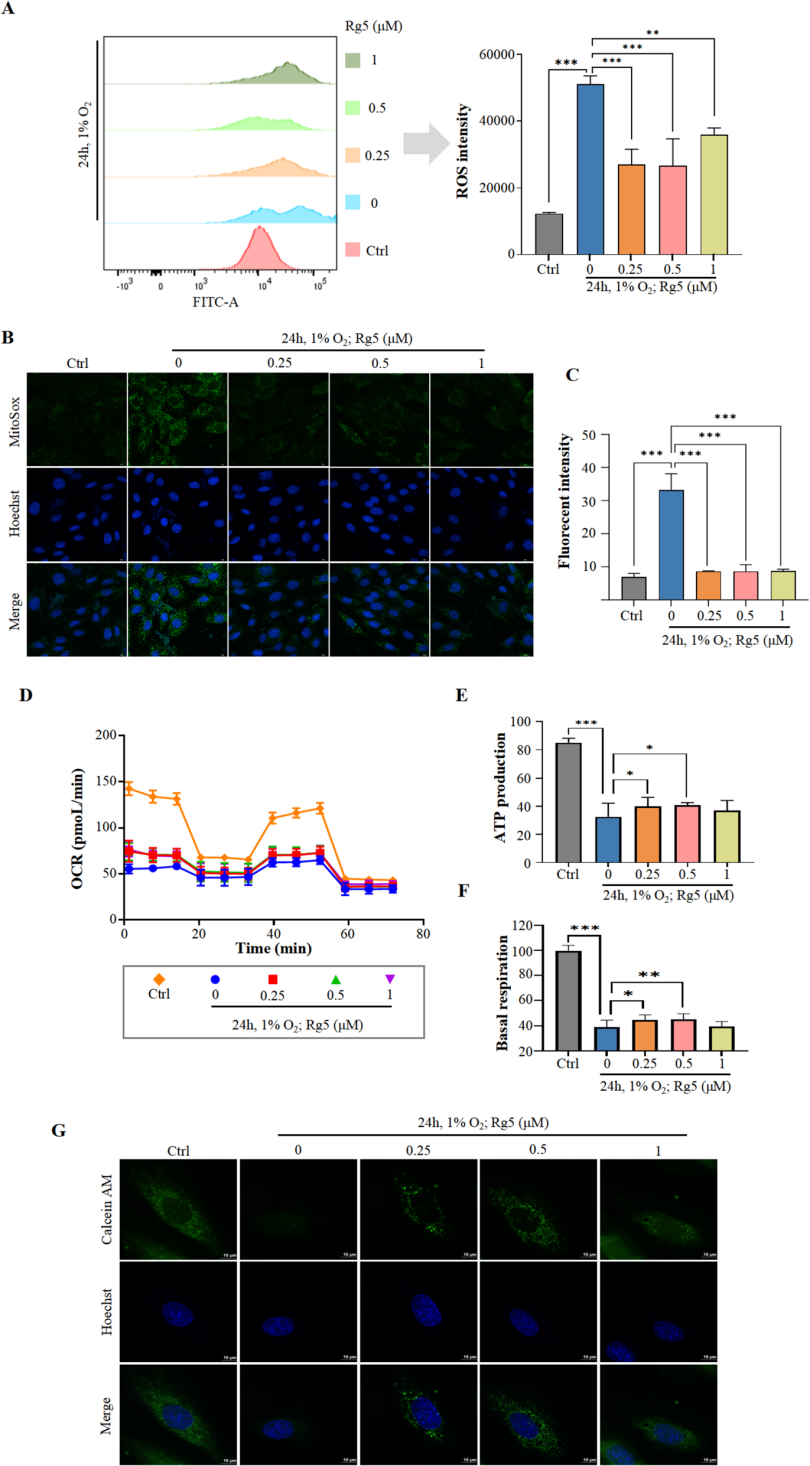
Rg5 mitigated oxidative stress and mitochondrial damage of H9c2 induced by acute hypoxia **A.** The ROS content in H9c2 cells was determined by flow cytometry (n = 3). **B, C.** MitoSOX dye was used to determine the content of ROS in mitochondria after treatment with Rg5 in H9c2 cells. Scale bar = 10 μm. **D**. The aerobic respiration capacity of H9c2 cells after hypoxia exposure was measured by a Seahorse XF Cell Mitochondrial Stress Test Kit. **E–F.** ATP production and basal respiration were quantified from Fig. 3D. **G**. Calcein AM dye was used to determine the MPTP after treatment with Rg5 in H9c2 cells. Scale bar = 10 μm. Data are shown as mean ± SD. *P < 0.05, **P < 0.01, ***P < 0.001 as indicated

Next, how Rg5 improved myocardial injury was explored. Flow cytometry results revealed that Rg5 reduced ROS levels in H9c2 cells after hypoxia exposure (Fig. 3A). MitoSOX dye, which allows us to observe the distribution of ROS in mitochondria, was used to further investigate the production of ROS in mitochondria after treatment with Rg5 in H9c2 cells. We found that similar to the overall ROS levels, there was an obviously decrease in mitochondrial ROS production of H9c2 cells after treatment with Rg5 (Fig. 3B, C). The production of ROS is generally associated with mitochondrial damage. To investigate the status of mitochondrial damage, we used the Seahorse XFe96 analyzer to detect mitochondrial function. The results showed that after hypoxia exposure, the basal respiration rate and ATP production rate of the cells significantly decreased, and treatment with Rg5 could improve these symptoms (Fig. 3D–F). Moreover, in the MPTP detection experiment, we found that the excessive opening of MPTP was significantly improved after Rg5 treatment (Fig. 3G). In summary, Rg5 protected mitochondrial function and reduce the production of ROS.

### Rg5 mitigates myocardial cell apoptosis caused by acute hypoxia

**Fig. 4:**
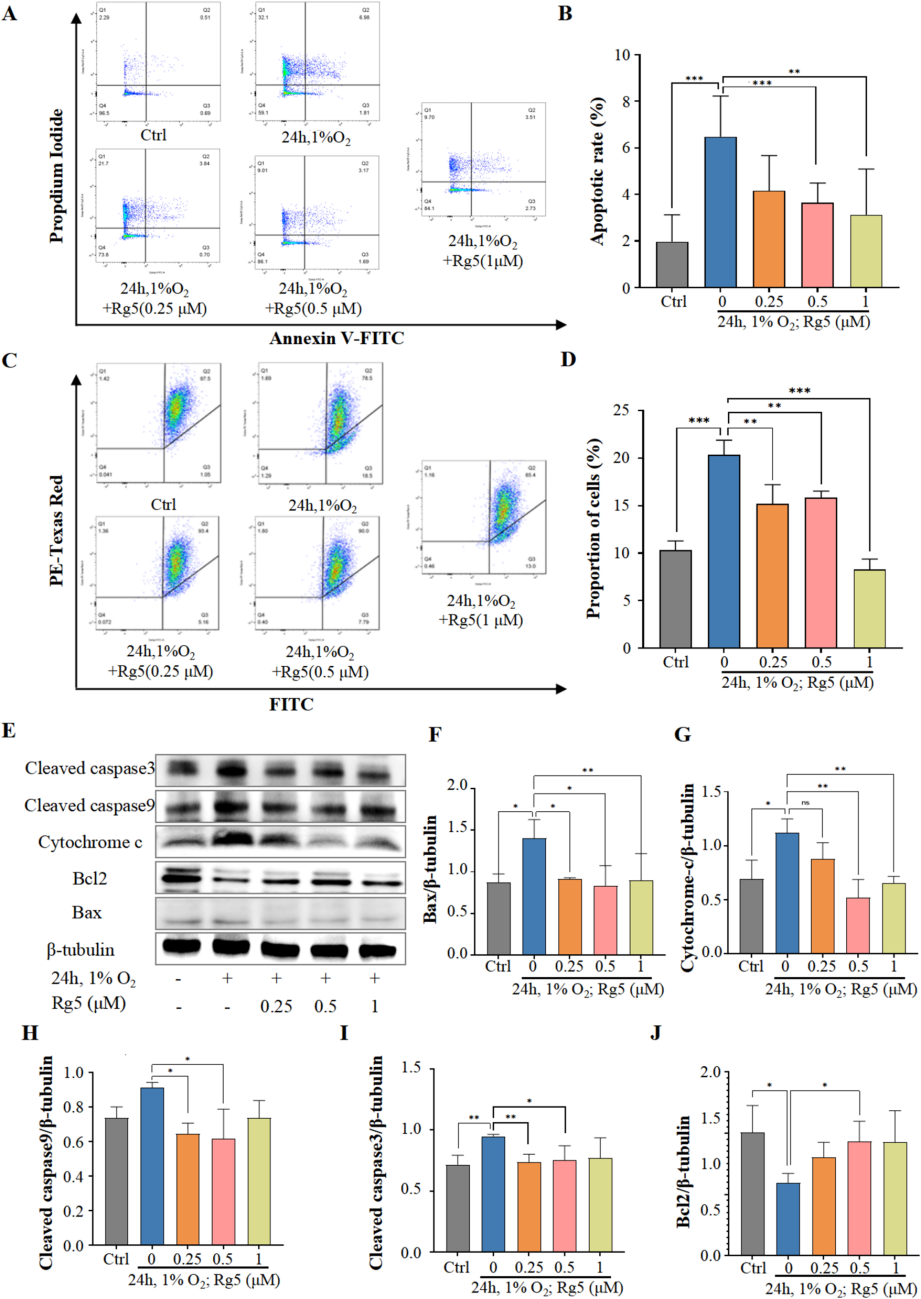
Rg5 mitigated the apoptosis of H9c2 cardiomyocytes induced by acute hypoxia. **A** Representative flow cytometry images using Annexin V/PI label to detect the hypoxia-induced apoptotic rate of H9c2 cells. **B** Statistical results of apoptotic rate detected by flow cytometry (n = 5). **C.** JC-1 mitochondrial membrane potential assay was performed using flow cytometry. **D** Quantitative comparison of mitochondrial membrane potential detected in Fig. 4c (n = 3). **E** Effect of Rg5 on apoptosis-related protein content in hypoxic H9c2 cardiomyocytes. **F-J.** WB quantitative results of apoptosis-related proteins: Bax (**F**), Cytochrome-c (**G**), Cytochrome-c (**H**), Cleaved caspase3 (**I**), Bcl2 (**J**). Data are shown as mean ± SD. *P < 0.05, **P < 0.01, ***P < 0.001 as indicated

Hypoxia-induced cell injury is commonly associated with apoptosis. To validate the relationship between apoptosis and Rg5, we performed flow cytometry using Annexin V/PI double labeling to detect the distribution of apoptotic cells induced by hypoxia. Flow cytometry analysis showed an increasing trend in the number of apoptotic cells among H9c2 cells exposed to hypoxia. However, in H9c2 cells pretreated with Rg5 before hypoxia exposure, the number of apoptotic cells markedly decreased (Fig. 4A, B). Changes in mitochondrial membrane potential also signal the onset of apoptosis, and the flow cytometry results of membrane potential detection were consistent with the apoptosis results, indicating that Rg5 could reduce the occurrence of membrane potential changes (Fig. 4C, D). Subsequently, we assessed the activation of the Bcl2 family in H9c2 cells treated with hypoxia through WB analysis (Fig. 4E). After treatment with Rg5, pro-apoptotic proteins such as Bax (Fig. 4F), cytochrome C (Fig. 4G), caspase-9 (Fig. 4H), and caspase-3 (Fig. 4I) were significantly reduced, while the anti-apoptotic protein Bcl2 (Fig. 4J) increased. These suggested that Rg5 might inhibit cell apoptosis via this pathway. These results indicated that Rg5 could inhibit apoptosis in H9c2 cells under hypoxic conditions.

### STAT3 is the key target of Rg5 in alleviating acute hypoxic myocardial apoptosis

**Fig. 5:**
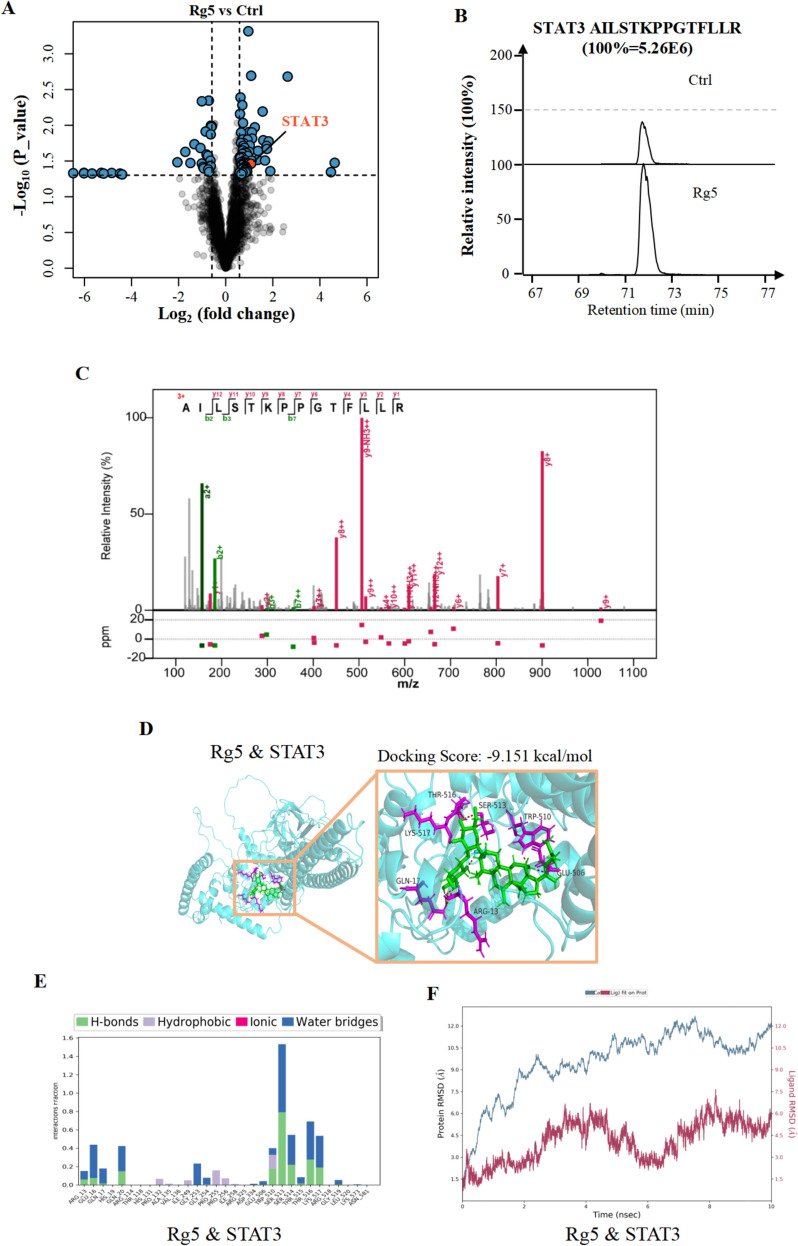
Evidence of Rg5 targeting STAT3 **A.** Thermal proteome screening data indicated that STAT3 was a potential target of Rg5. **B.** Schematic diagram of peak intensity of a representative peptide of STAT3 in the thermal proteome data. **C.** STAT3 peptide AILSTKPPGTFLLR was identified with good confidence. **D.** Molecular docking results showed that Rg5 and STAT3 had good binding ability. **E** Schematic diagram of binding sites for molecular docking of Rg5 & STAT3. **F** Schematic diagram of binding energy between Rg5 and STAT3

**Fig. 6:**
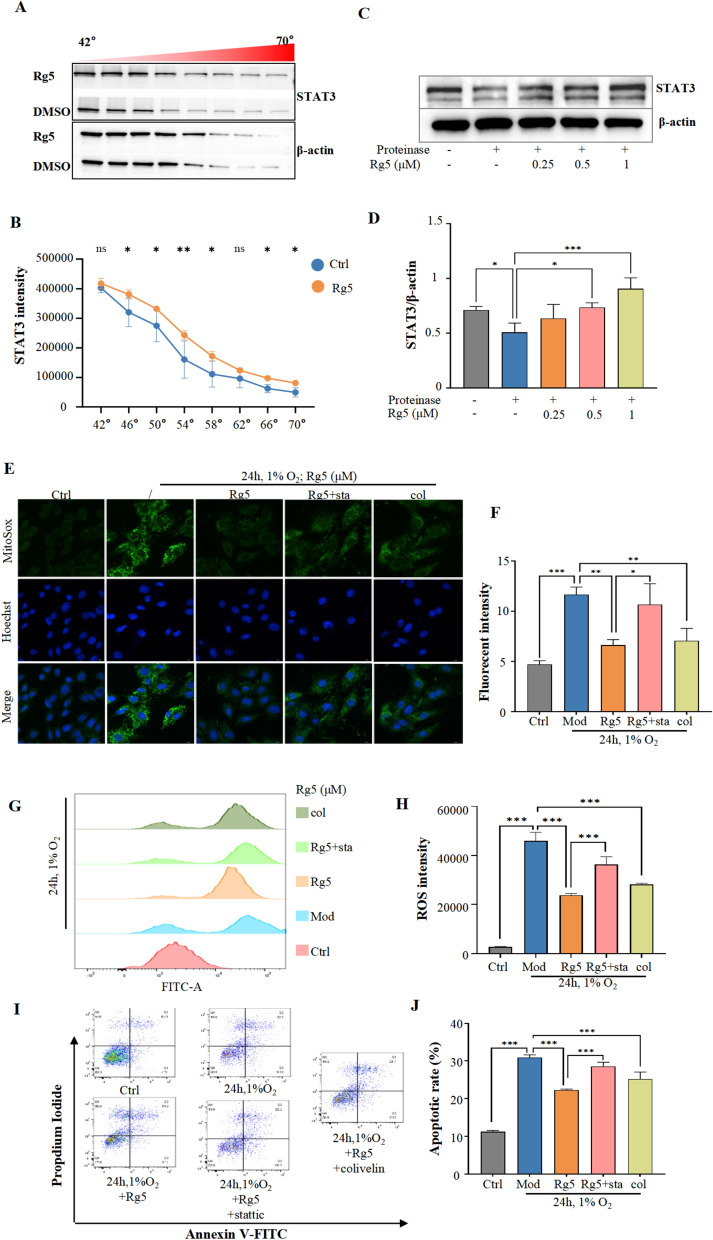
STAT3 is the key target of Rg5 in alleviating acute hypoxic myocardial injury. **A** Cellular thermal shift assay (CETSA) showed that the degradation of STAT3 slowed down after Rg5 treatment. **B** WB quantitative results of CESTA. **C** The degradation of STAT3 was hindered by Rg5 in Drug affinity responsive target stability (DARTS) assay. **D** WB quantitative results of DARTS. **E–F.** MitoSOX dye was used to determine the content of ROS in mitochondria after treatment with Rg5, Rg5 + stattic (sta) and colibelin (col) in H9c2 cells. Scale bar = 10 μm. **G.** The ROS content in H9c2 cells was determined by flow cytometrya. **H.** The statistical quantification of ROS content based on Fig. 6E (n = 3). **I.** Representative flow cytometry images using Annexin V/PI label to detect the effects of STAT3 inhibitors and agonists on apoptotic rate. **J.** Statistical results of apoptotic rate detected by flow cytometry (n = 3). Data are shown as mean ± SD. ***P < 0.001 as indicated

In order to elucidate the specific mechanism by which Rg5 reduces acute hypoxic myocardial apoptosis, we firstly used thermal proteomics techniques to screen potential target proteins of Rg5 (Fig. 5A). Among the potential target proteins, signal transducer and activator of transcription 3 (STAT3) has attracted our attention. The thermal proteome results showed that after the treatment of Rg5, the peptide content of STAT3, such as AILSTKPPGTFLLR, was obviously up-regulated (Fig. 5B), and the spectrum of this peptide also showed clear identification (Fig. 5C). In addition, molecular docking was also performed to evaluate the binding ability of Rg5 and STAT3, and the results showed that the affinity reached -9.151 kcal/mol (Fig. 5D). Then we found that Rg5 does not directly bind to the SH2 domain, but binds to nearby sites. For example, serine at position 513 (Fig. 5E). According to the energy data graph, Rg5 and STAT3 can be firmly combined (Fig. 5F). Therefore, the theoretical results showed that there was a strong interaction between Rg5 and STAT3. Additionally, cellular thermal shift assay (CETSA) showed that Rg5 could significantly slow down the degradation of STAT3 caused by temperature increase (Fig. 6A, B). Drug affinity responsive target stability (DARTS) experiments also showed that Rg5 could retard the degradation of STAT3 (Fig. 6B, C). All the above results proved that Rg5 could target STAT3.

Next, we demonstrated that Rg5 interacts with STAT3 functionally. Stattic, an inhibitor of STAT3, could significantly reverse the reduction of ROS level in mitochondria of H9c2 cardiomyocytes induced by Rg5 (Fig. 6E, F). Moreover, compared with Rg5 group, the overall intracellular ROS level was also significantly increased after stattic treatment by flow cytometry (Fig. 6G, H). At the same time, we found that the STAT3 agonist colivelin has the effect of reducing the content of intracellular ROS, which showed the same effect as Rg5. Focusing on apoptosis, the inhibitor stattic significantly increased the occurrence of apoptosis compared to Rg5 group. On the contrary, colivelin could significantly decrease the occurrence of apoptosis compared with Mod group (Fig. 6I, J). These results indicated that Rg5 not only "physically" directly interacted with STAT3, but also "functionally" promoted STAT3 activity.

### Rg5 alleviates hypoxia-induced myocardial apoptosis by promoting Tyr705 phosphorylation of STAT3

**Fig. 7:**
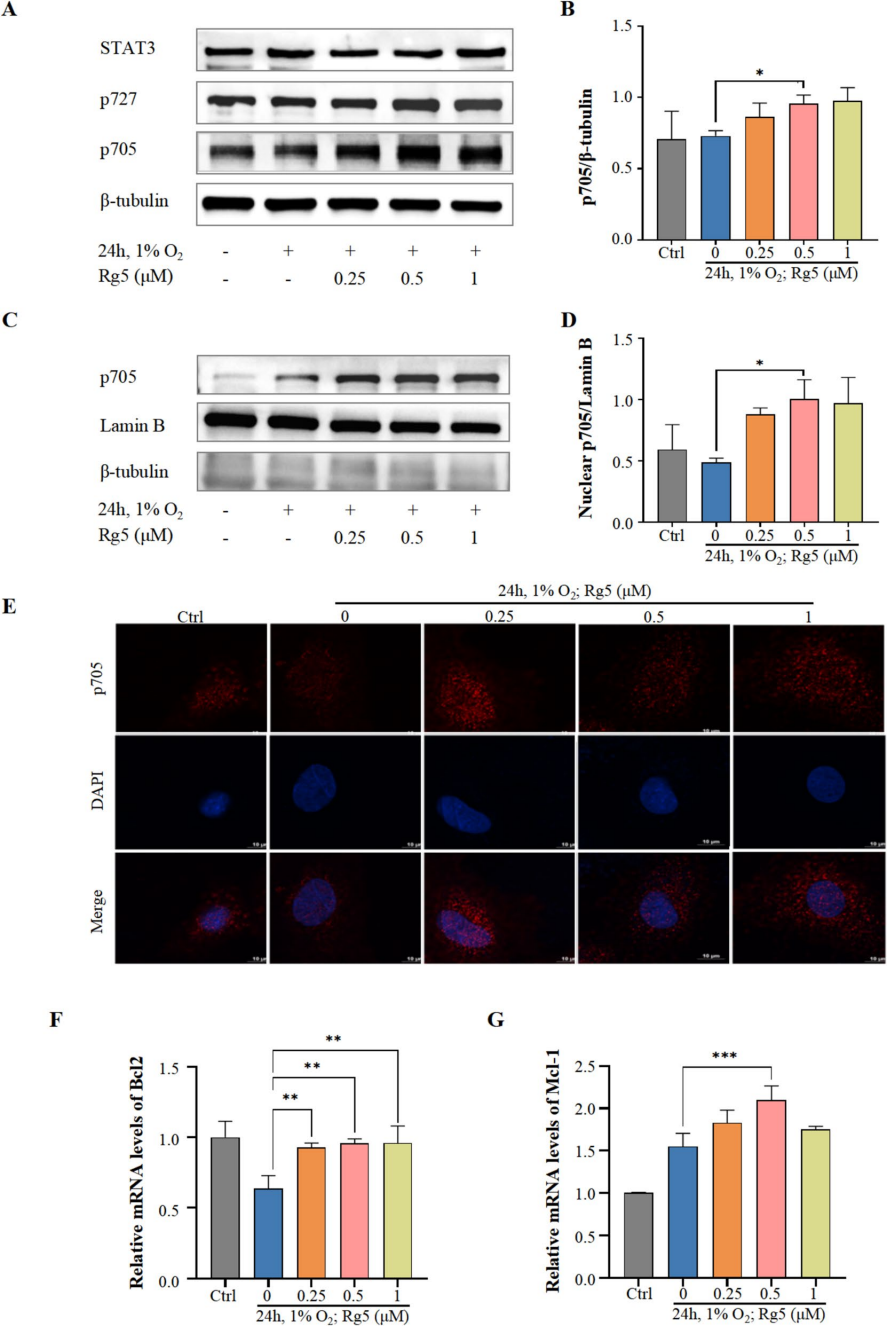
Rg5 enhanced phosphorylation of STAT3 Tyr705 and promoted nuclear transfer of STAT3 **A.** Rg5 specifically promoted the phosphorylation of Stat3 Tyr705 in whole cell lysate. **B** The statistical quantification of Try705 phosphorylation (p705) based on Fig. 7a (n = 3). **C.** WB results of p705 in the nucleus after Rg5 treatment. **D.** The content changes of p705 in nucleus calculated based on Fig. 7c (n = 3). **E.** Immunofluorescence results showed that Rg5 promoted the aggregation of p705 in the nucleus. Scale bar = 10 μm. **F-G.** mRNA levels of the target proteins STAT3 transcriptionally regulated (n = 3). Bcl2 (**F**), Mcl-1 (**G**). Data are shown as mean ± SD. *P < 0.05, **P < 0.01, ***P < 0.001 as indicated

STAT3 is an important signal transduction protein, and its activity is mainly regulated by the phosphorylation levels at 705 tyrosine and 727 serine. WB results showed that Rg5 significantly increased the phosphorylation level of STAT3 Tyr705 in H9c2 cardiomyocytes after hypoxia exposure (Fig. 7A, B), but had no effect on the protein content of STAT3 and phosphorylation at Ser727. Phosphorylation of STAT3 at Try705 can mediate the transfer of STAT3 to the nucleus [25]. By isolating the nucleus, it was found that STAT3 phosphorylated at Tyr705 increased significantly in the nucleus after the administration of Rg5 (Fig. 7C-E). At the same time, we detected downstream target proteins of STAT3, Bcl2 and Mcl-1. RT-qPCR results showed that Rg5 promoted the transcriptional activation of Bcl2 (Fig. 7F) and Mcl-1(Fig. 7G). These results suggested that Rg5 could exert the pharmacodynamic effect by activating Tyr705 phosphorylation of STAT3.

**Fig. 8:**
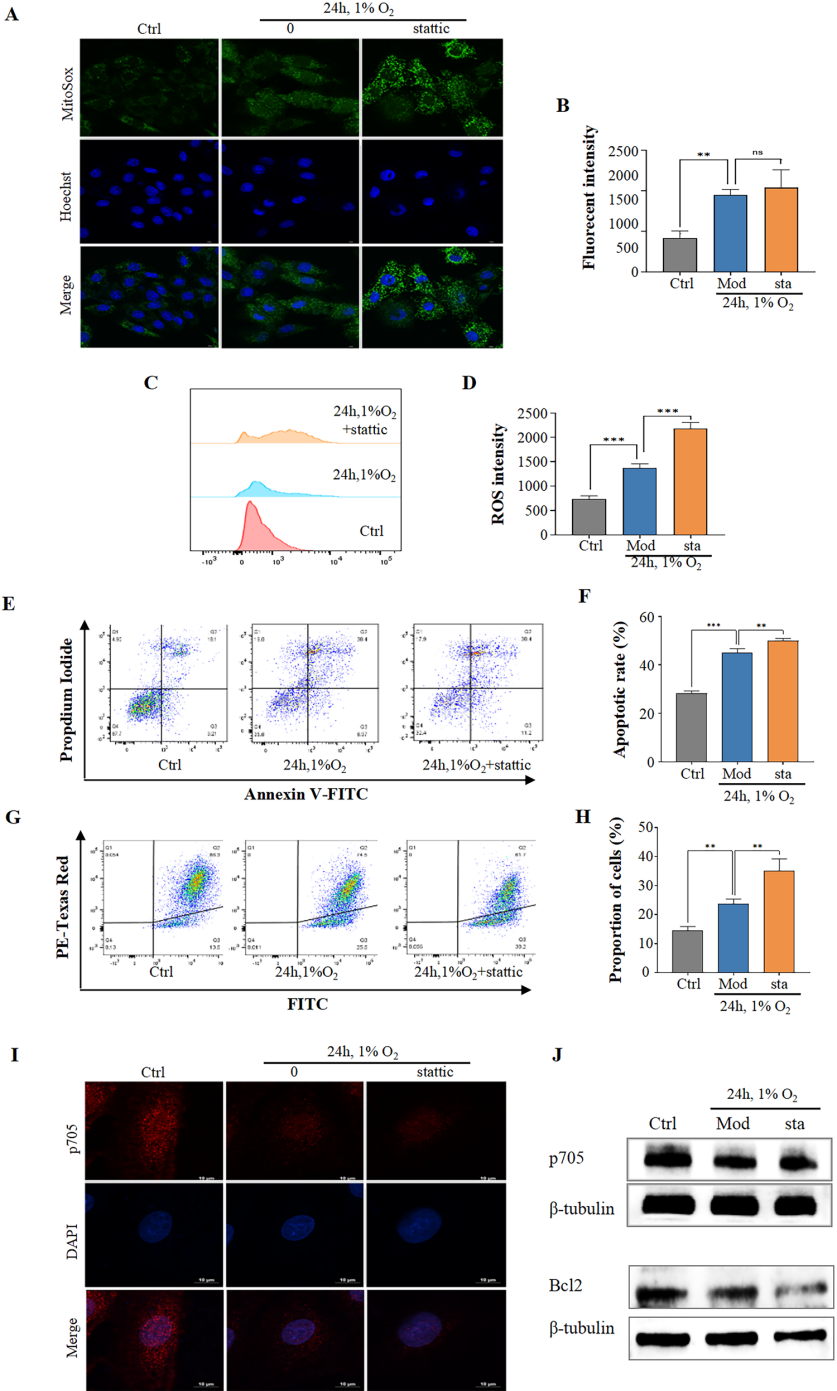
Stattic, a STAT3 p705-specific inhibitor, exacerbated hypoxic-induced apoptosis of H9c2 cardiomyocytes **A-B.** MitoSOX staining results showed that stattic (sta) further increased the accumulation of ROS caused by hypoxia. Scale bar = 10 μm. **C, D.** The ROS content in H9c2 cells was determined by flow cytometry after sta treatment (n = 3). **E–F.** Annexin V/PI results detected by flow cytometry showed that sta increased the apoptotic rate of H9c2 cardiomyocytes compared with Mod group (n = 3). **G-H.** Sta caused more drastic changes in mitochondrial membrane potential compared with Mod group (n = 3). **I.** Sta inhibited the accumulation of STAT3 p705 in the nucleus. Scale bar = 10 μm. **J.** Sta inhibited the content of STAT3 p705 compared with the Mod group. Data are shown as mean ± SD. **P < 0.01, ***P < 0.001 as indicated

Furthermore, we found that stattic significantly increased ROS levels in hypoxic-treated H9c2 cardiomyocytes (Fig. 8A–D). Correspondingly, stattic further promoted the apoptosis of H9c2 cardiomyocytes after hypoxia exposure (Fig. 8E, F), which was also confirmed by a dramatic change in mitochondrial membrane potential (Fig. 8G, H). In addition, the level of phosphorylated STAT3 at Tyr705 in the nucleus was further reduced after stattic treatment compared with the group which was treated by hypoxia (Fig. 8I). Meanwhile, the level of phosphorylated STAT3 at Tyr705 in the whole cell was also significantly decreased (Fig. 8J).

### Rg5 enhances the interaction between STAT3 and JAK2

**Fig. 9:**
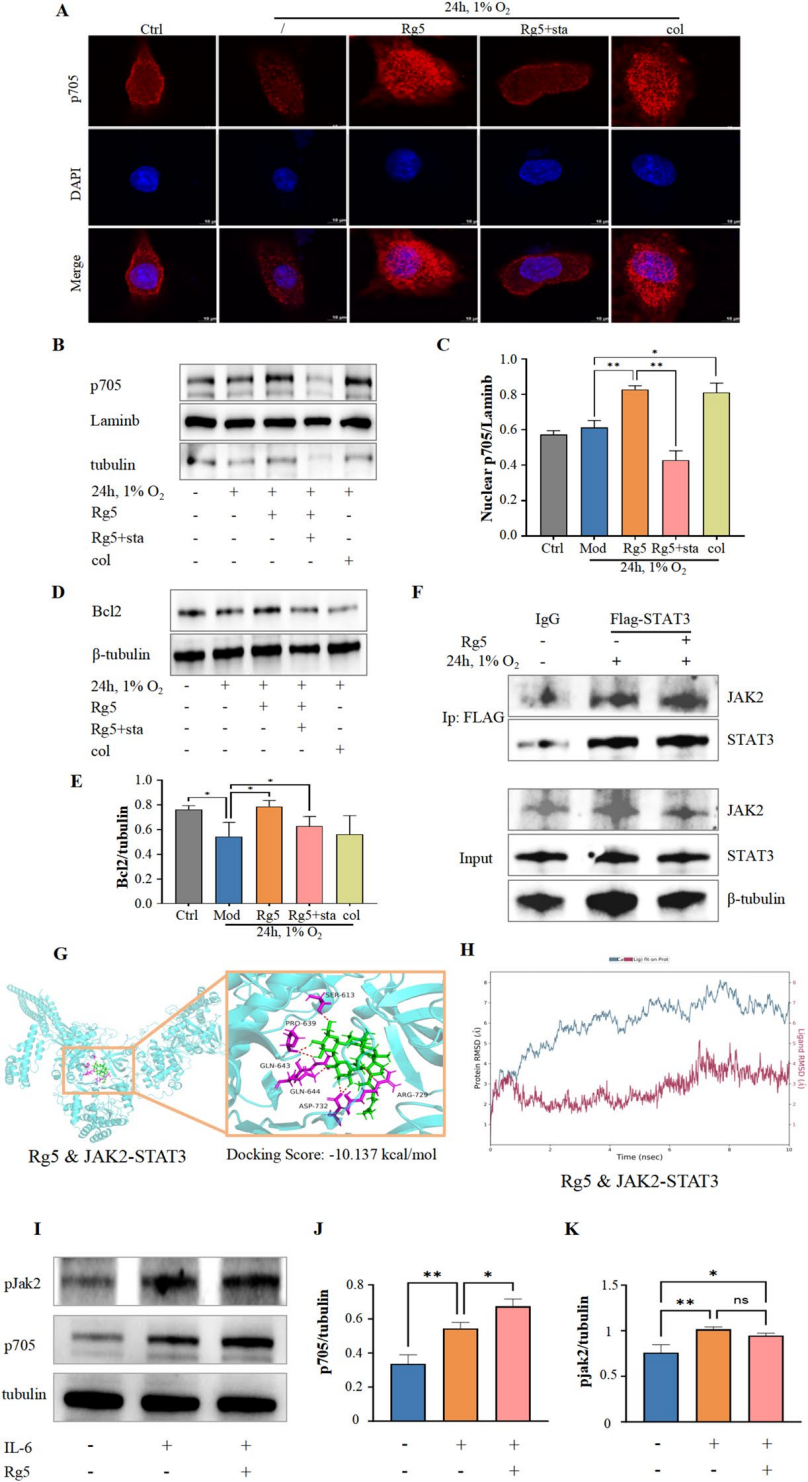
Stattic reversed the improvement of Rg5 on the apoptosis of H9c2 cardiomyocytes induced by acute hypoxia **A.** Immunofluorescence results showed that the accumulation of STAT3 p705 in the nucleus was significantly reduced after Stattic intervention compared to the Rg5 group. Scale bar = 10 μm. **B.** WB results of STAT3 p705 in nucleus of H9c2 cardiomyocytes. **C.** Sta significantly reduced the amount of STAT3 p705 in the nucleus compared to Rg5 group, as calculated in Fig. 3b (n = 3). **D.** WB results of Bcl2 in H9c2 cardiomyocytes treated by sta. **E.** Sta significantly reduced the amount of Bcl2 compared to Rg5 group, as calculated in Fig. 4j (n = 3). **F.** Immunoprecipitation results showed that Rg5 enhanced the interaction between JAK2 and STAT3. **G**. Molecular docking results showed that Rg5, STAT3, JAK2 had good binding ability. **H** Schematic diagram of binding energies of Rg5, STAT3, JAK2. **I**. WB results of p705 and pJAK2 in H9c2 cardiomyocytes treated by IL-6. WB quantitative results of proteins: Bax (**J**), Cytochrome-c (**K**). Data are shown as mean ± SD. *P < 0.05, **P < 0.01 as indicated

Subsequently, we further verified the regulation of Rg5 on Tyr705 phosphorylation of STAT3 through STAT3 agonists and inhibitors. Firstly, immunofluorescence results showed that the inhibitor stattic did significantly reduce the accumulation of STAT3 phosphorylated at Tyr705 in the nucleus compared with Rg5 group (Fig. 9A). Colivelin, the agonist of STAT3, significantly promoted STAT3 accumulation in the nucleus compared with Mod group. WB results of separated nuclear components also showed that the content of STAT3 phosphorylated at Tyr705 significantly decreased after the administration of stattic compared with Rg5 group (Fig. 9B, C), while the content of STAT3 phosphorylated at Tyr705 in the nucleus was significantly increased by colivelin. The downstream anti-apoptotic protein Bcl2 also showed the expected effect, which was significantly reduced after the administration of stattic compared with Rg5 group (Fig. 9D, E). Then, through molecular docking and immunoprecipitation experiments, it was confirmed that the binding ability of STAT3 and JAK2, the kinase of STAT3, was significantly enhanced during Rg5 exposure (Fig. 9F–H). Finally, the WB experiment results showed that Rg5 promoted STAT3 phosphorylation without affecting JAK2 in the presence of IL-6 activation (Fig. 9I–K). These results indicated that Rg5 could enhance the interaction between STAT3 and JAK2, and further promoted the phosphorylation of Tyr705 of STAT3.

### Inhibition of Stat3 reduces the protective effect of Rg5

**Fig. 10:**
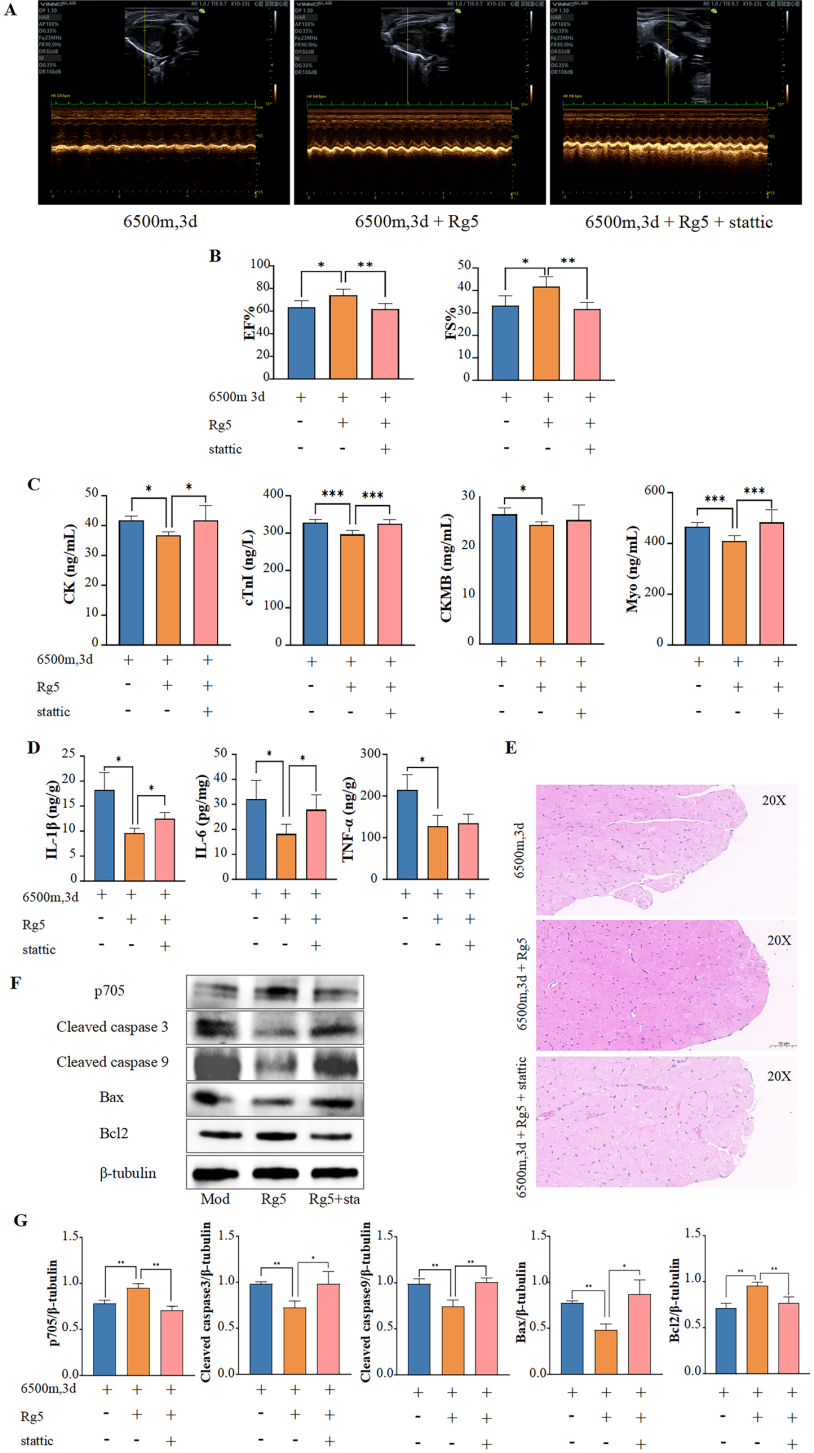
Rg5 improved acute hypoxic heart injury by targeting STAT3 in vivo **A.** Representative echocardiographic image of mice in three groups. **B** Sta reversed Rg5’s improvement in cardiac function of hypoxic mice. EF, ejection fraction and FS, left ventricular fractional shortening (n = 6). **C** Content changes of myocardial injury markers in the peripheral blood affect by sta. CK, Creatine kinase; cTnI, Cardiac troponin I; CK-MB, Creatine kinase-MB; Myo, Myoglobin (n = 6). **D** Content changes of IL-1β, IL-6, and TNF-α in peripheral blood affected by sta (n = 6). **E** Representative HE staining image of heart tissues in three group of mice. Scale bar = 50 μm. **F** WB results of STAT3 p705 and apoptosis-related proteins of mouse hearts. **G** Content changes of STAT3 p705 and apoptosis-related proteins based on Fig. 10g (n = 3). Data are shown as mean ± SD. *P < 0.05, **P < 0.01, ***P < 0.001 as indicated

After elucidating the relationship between Rg5 and STAT3 in vitro, we attempted to explore whether stattic would reverse the pharmacological effects of Rg5 in vivo. Three groups were set up: Hypoxia group, Rg5 group and Rg5 + stattic group. All mice were exposed to a hypoxic environment. Firstly, echocardiography results showed that the ejection fraction and fractional shortening of mouse hearts were significantly reduced in Rg5 + stattic group compared with Rg5 group (Fig. 10A, B). The levels of myocardial enzymes in serum including CK, cTnI, and Myo were significantly increased in Rg5 + stattic group compared with Rg5 group (Fig. 10C), while the content of CKMB also showed an increasing trend, but there was no statistical difference due to large fluctuations. At the same time, there were also increased inflammatory response in mouse heart of Rg5 + stattic group (Fig. 10D). Furthermore, HE staining results indicated that while Rg5 was able to alleviate the edema in hypoxic cardiomyocytes, this protective effect was significantly diminished after stattic administration, with the mouse hearts displaying obvious edema similar to the Hypoxia group (Fig. 10E). Finally, we reconfirmed in mouse heart tissue by WB that Rg5 promoted the phosphorylation of STAT3 at tyrosine 705, while stattic reversed this increase. Additionally, the levels of apoptosis-related proteins were also significantly reversed after stattic administration compared to the Rg5 group (Fig. 10F, G). These results all suggest that the pharmacological effects of Rg5 in vivo is also achieved by promoting STAT3 phosphorylation.

## Discussion

Acute exposure to hypoxic environment at high altitude often causes fluster, shortness of breath, fatigue and other symptoms [26]. As the pumping organ of the body, the heart tissue can relieve the challenge of hypoxia by overload operation. However, since the adult mammalian heart does not have regenerative capacity [6], hypobaric hypoxia can cause irreversible damage. In this study, it was found that hypoxia can lead to decreased cardiac function, edema of cardiac cells in vivo, and increase mitochondrial oxidative stress and apoptosis in vitro. These findings are consistent with previous reports [5, 27, 28]. Meanwhile, we also found in vivo that there was no obvious apoptosis in the mouse heart, such as the green fluorescence of TUNEL staining was not obvious (data not shown), which might be due to insufficient hypoxia exposure or animal strain perturbation. Wang et al. reported that cardiac tissue of Sprague–Dawley (SD) rats showed significant apoptosis after hypoxia exposure for 21 days at 5500 m [29].

Ginseng, as a highly respected holy product for invigorating “qi” since ancient times, plays a pivotal role in the clinical practice of traditional Chinese medicine [30, 31]. Red ginseng is one of the best, containing rich active ingredients, among which Rg5 has become a research hotspot in recent years because of its unique biological activity and pharmacological effect [32, 33]. Acute mountain sickness often presents "qi deficiency" symptoms. Therefore, it is of far-reaching significance to explore the efficacy of Rg5 in improving altitude sickness. This study showed that Rg5 could significantly reduce the structural and functional damage of the heart caused by hypoxic exposure in vivo. Further, it was found that Rg5 could effectively reverse mitochondrial damage, oxidative stress and apoptosis under hypoxic conditions, and improve the decline of cell vitality caused by hypoxia. These findings are consistent with previous reports [34, 35] that Rg5 could alleviate oxidative stress and apoptosis under specific conditions, further consolidating the potential of Rg5 as an antioxidant and anti-apoptosis to ameliorate hypoxic heart injury. It is particularly worth mentioning that Rg5 also showed significant anti-inflammatory activity, which is consistent with the conclusions of Lee et al. [36]. Inflammation is an important pathological process [37, 38] of altitude diseases, and relieving the inflammatory response is very important for the treatment and rehabilitation of altitude diseases.

In addition, Rg5 also demonstrates excellent therapeutic efficacy in the treatment of other hypoxic diseases and cardiac diseases, thanks to its anti-inflammatory, anti-apoptotic and anti-hypoxic activities. Existing studies have confirmed that Rg5 can inhibit Ang II-induced cardiac inflammation and remodeling [39]. In addition, Rg5 has been proven to alleviate the endoplasmic reticulum stress response and inhibit the activation of NLRP3 inflammasome [40]. This mechanism not only has protective significance for hypoxic cardiomyocytes but may also provide new intervention strategies for hypoxic injury in other organs. It is notable that recent studies have also found that Rg5 can inhibit the increase in Hif-1α level caused by hypoxia, showing therapeutic potential for erectile dysfunction [41]. Additionally, long-term hypoxia exposure often leads to compensatory hypertrophy of the heart, which is closely related to excessive apoptosis and inflammatory responses [42, 43]. Based on the excellent pharmacological activities of Rg5 in anti-apoptosis and reducing inflammatory responses, we speculate that Rg5 may also have ameliorative effect on cardiac hypertrophy and hypertrophic cardiomyopathy (HCM), although this requires substantial evidence. Therefore, the various pharmacological effects of Rg5 provide a promising strategy for alleviating hypoxic diseases and heart diseases.

Mass spectrometry-based drug target protein discovery technology has shown extensive application potential in the field of drug development [44–46]. In this study, the combination of thermal stability and mass spectrometry method has innovatively revealed that Rg5 may target STAT3. Although STAT3 was not the most significant candidate target during the initial data screening, combined with the results of network pharmacology, we chose to focus on STAT3. Meanwhile, in view of its central role and extensive functions in cell signal transduction, we conducted a series of cellular experiments in depth, which strongly confirmed that Rg5 indeed directly target STAT3. Rg5 not only has a stable interaction with STAT3 at the physical level, but is also functionally closely connected to STAT3. Specifically, we found that Rg5 could significantly mitigate the apoptosis of H9c2 cardiomyocytes under hypoxia conditions, and this effect was mediated by STAT3, which was mainly confirmed by STAT3 inhibitor. Since STAT3 is present in both mitochondria and nuclear [47], in order to reveal the biological effects of Rg5 binding to STAT3, we further isolated mitochondrial (results not shown) and nuclear components. Interestingly, we found that Rg5 was able to promote STAT3 accumulation in the nucleus, but not in the mitochondria. It has been reported that STAT3 nuclear transfer can activate the expression of a series of anti-apoptosis-related proteins [48]. In this study, we also observed a similar phenomenon. Rg5 increases the expression of anti-apoptotic proteins BCL2 and MCL1 by promoting STAT3 nuclear transfer. It is worth mentioning that BCL2 is also an important regulatory factor of mitochondrial membrane permeability transition pore (MPTP), which affects the opening of MPTP and subsequently affects the production of intracellular ROS [49]. Studies have confirmed that Rg5 can control the excessive opening of MPTP [14]. Rg5 may reduce cell apoptosis through BCL2 while also controlling MPTP through BCL2, thereby reducing ROS production in cells after hypoxia exposure. These findings not only further confirm the hypothesis that Rg5 plays pharmacological effects by targeting STAT3 and promoting its nuclear transfer, but also provide a new perspective and mechanism for further understanding of the myocardial protective effect of Rg5.

The nuclear localization of STAT3 is mainly mediated by Tyr705 phosphorylation [25], in which JAK2 kinase is responsible for the phosphorylation of Tyr705 and plays an important role in this process [50, 51]. We found that Rg5 specifically promoted the phosphorylation of STAT3 at Tyr705, but had no significant effect on the phosphorylation of STAT3 at Ser727. Most reports about the STAT3 Tyr705 focus on proliferation, differentiation, and immunosuppression functions [47, 52]. In addition, STAT3 Try705 is the key anti-tumor site [53]. This study did not focus on the effect of STAT3 on the proliferation of tumor, but on whether myocardial damage in hypoxia was improved by the activation of STAT3. As expected, we found that hypoxia caused cardiomyocyte apoptosis and affected cardiac function maintenance in mice. However, after the treatment of Rg5, both in vivo and in vitro, Rg5 played a protective role by activating the phosphorylation of STAT3 Tyr705. Through MD, WB, IP experiments, we found that Rg5 activated Tyr705 phosphorylation by promoting the interaction between JAK2 and STAT3. Furthermore, we discovered that Rg5 could enhance the phosphorylation level of STAT3 Tyr705 without affecting the phosphorylation of JAK2. This also suggested that Rg5 could promote the binding between STAT3 and JAK2.

Although this study reveals that Rg5 can target STAT3, and promote phosphorylation of STAT3 Tyr705 to improve hypoxic myocardial injury, there are still some limitations. We drew on previous research achievements [54, 55] and ultimately decided to evaluate the in vivo efficacy of Rg5 through oral administration at a dose of 30 mg/kg. However, it is inevitable that oral Rg5 intake affects the intestinal microbiota, which is closely associated with the myocardial injury [56–58]. Additionally, studies have reported that Rg5 can promote angiogenesis by targeting IGF-1, thereby improving ischemic injury [59], which may also contribute to the amelioration of hypoxic myocardial injury. Consequently, we cannot rule out these possible molecular mechanisms mentioned above. In addition, Rg5 did not affect the content of STAT3, but enhanced the interaction of JAK2 and STAT3, and promoted the phosphorylation level of Tyr705. So, what is the structural basis of the interaction of Rg5, JAK2 and STAT3? Although we have preliminarily explored the possible structure through molecular docking and molecular dynamics simulations, it is still necessary to elucidate the precise structure of this complex via relevant molecular biology experiments or techniques such as cryo-electron microscopy.

## Conclusions

In summary, this study reveals that acute hypoxia exposure leads to impaired cardiac function, which is manifested by increased cardiomyocyte apoptosis, inflammatory response, and elevated oxidative stress. Rg5, a natural product from ginseng, can improve the cardiac function of mice exposed to hypoxia and reduce myocardial apoptosis. The specific mechanism is that Rg5 promotes the interaction of JAK2 and STAT3 by targeting STAT3 and induces the phosphorylation of STAT3 at tyrosine 705, which alleviates apoptosis and enhance the viability of cardiomyocytes. This study elucidates the pharmacological effects and molecular mechanism of Rg5 in mitigating acute hypoxic myocardial injury, providing a potential therapeutic option for hypoxia-induced myocardial injury.

## Data Availability

Data will be made available on request.

## Abbreviations

CK: Creatine kinase
CKMB: Creatine kinase-myocardial band
cTnI: Cardiac troponin I
DMEM: Dulbecco's modified eagle medium
DMSO: Dimethyl sulfoxide
EF: Ejection fraction
FBS: Fetal bovine serum
FS: Fractional shortening
GSH: Glutathione
IL-1β: Interleukin-1 beta
IL-6: Interleukin-6
JAK2: Janus kinase 2
MAPK: Mitogen-activated protein kinase
MDA: Malondialdehyde
MPTP: Mitochondrial permeability transition pore
MYO: Myoglobin
PBS: Phosphate-buffered saline
PKC: Protein Kinase C
ROS: Reactive oxygen species
SOD: Superoxide dismutase
STAT3: Signal transducer and activator of transcription 3
TNF-α: Tumor Necrosis Factor-Alpha
